# Switching to dual/monotherapy determines an increase in CD8+ in HIV-infected individuals: an observational cohort study

**DOI:** 10.1186/s12916-018-1046-2

**Published:** 2018-05-29

**Authors:** Cristina Mussini, Patrizia Lorenzini, Alessandro Cozzi-Lepri, Giulia Marchetti, Stefano Rusconi, Andrea Gori, Silvia Nozza, Miriam Lichtner, Andrea Antinori, Andrea Cossarizza, Antonella d’Arminio Monforte, for the Icona Foundation Study Group, A. d’Arminio Monforte, A. Antinori, A. Castagna, F. Castelli, R. Cauda, G. Di Perri, M. Galli, R. Iardino, G. Ippolito, A. Lazzarin, G. C. Marchetti, C. F. Perno, G. Rezza, F. von Schloesser, P. Viale, F. Ceccherini-Silberstein, A. Cozzi-Lepri, E. Girardi, S. Lo Caputo, C. Mussini, M. Puoti, M. Andreoni, A. Ammassari, C. Balotta, A. Bandera, P. Bonfanti, S. Bonora, M. Borderi, A. Calcagno, L. Calza, M. R. Capobianchi, A. Cingolani, P. Cinque, A. De Luca, A. Di Biagio, N. Gianotti, A. Gori, G. Guaraldi, G. Lapadula, M. Lichtner, G. Madeddu, F. Maggiolo, G. Marchetti, S. Marcotullio, L. Monno, S. Nozza, E. Quiros Roldan, R. Rossotti, S. Rusconi, M. M. Santoro, A. Saracino, M. Zaccarelli, I. Fanti, L. Galli, P. Lorenzini, A. Rodano, M. Shanyinde, A. Tavelli, F. Carletti, S. Carrara, A. Di Caro, S. Graziano, F. Petrone, G. Prota, S. Quartu, S. Truffa, A. Giacometti, A. Costantini, V. Barocci, G. Angarano, C. Santoro, C. Suardi, V. Donati, G. Verucchi, C. Minardi, T. Quirino, C. Abeli, P. E. Manconi, P. Piano, B. Cacopardo, B. Celesia, J. Vecchiet, K. Falasca, L. Sighinolfi, D. Segala, P. Blanc, F. Vichi, G. Cassola, C. Viscoli, A. Alessandrini, N. Bobbio, G. Mazzarello, C. Mastroianni, I. Pozzetto, I. Caramma, A. Chiodera, P. Milini, G. Rizzardini, M. C. Moioli, R. Piolini, A. L. Ridolfo, S. Salpietro, C. Tincati, C. Puzzolante, N. Abrescia, A. Chirianni, G. Borgia, R. Orlando, G. Bonadies, F. Di Martino, I. Gentile, L. Maddaloni, A. M. Cattelan, S. Marinello, A. Cascio, C. Colomba, F. Baldelli, E. Schiaroli, G. Parruti, F. Sozio, G. Magnani, M. A. Ursitti, A. Cristaudo, V. Vullo, R. Acinapura, G. Baldin, M. Capozzi, S. Cicalini, L. Fontanelli Sulekova, G. Iaiani, A. Latini, I. Mastrorosa, M. M. Plazzi, S. Savinelli, A. Vergori, M. Cecchetto, F. Viviani, P. Bagella, B. Rossetti, A. Franco, R. Fontana Del Vecchio, D. Francisci, C. Di Giuli, P. Caramello, G. C. Orofino, M. Sciandra, M. Bassetti, A. Londero, G. Pellizzer, V. Manfrin, G. Starnini, A. Ialungo

**Affiliations:** 1 Clinic of Infectious Diseases, University Hospital, University of Modena and Reggio Emilia, Via del Pozzo, 71, 41124 Modena, Italy; 2 National Institute for Infectious Diseases L. Spallanzani, Rome, Italy; 3 Department of Infection and Population Health Division of Population Health, University College London, Hampstead Campus, London, UK; 4 Clinic of Infectious Diseases, Department of Health Sciences San Paolo Hospital, DIBIC Luigi Sacco, University of Milan, Milan, Italy; 5 Clinic of Infectious Diseases, San Raffaele Hospital, University Vita e Salute, Milan, Italy; 6 Department of Public Health and Infectious Diseases, Sapienza University of Rome, Polo Pontino, Italy; 7 Pathology and Immunology, University of Modena and Reggio Emilia, Modena, Italy

**Keywords:** CD8, CD4/CD8 ratio, Chronic inflammation, Monotherapy, Dual therapy

## Abstract

**Background:**

The CD4/CD8 ratio has been associated with the risk of AIDS and non-AIDS events. We describe trends in immunological parameters in people who underwent a switch to monotherapy or dual therapy, compared to a control group remaining on triple antiretroviral therapy (ART).

**Methods:**

We included patients in Icona who started a three-drug combination ART regimen from an ART-naïve status and achieved a viral load ≤ 50 copies/mL; they were subsequently switched to another triple or to a mono or double regimen. Standard linear regression at fixed points in time (12-24 months after the switch) and linear mixed model analysis with random intercepts and slopes were used to compare CD4 and CD8 counts and their ratio over time according to regimen types (triple vs. dual and vs. mono).

**Results:**

A total of 1241 patients were included; 1073 switched to triple regimens, 104 to dual (72 with 1 nucleoside reverse transcriptase inhibitor (NRTI), 32 NRTI-sparing), and 64 to monotherapy. At 12 months after the switch, for the multivariable linear regression the mean change in the log_10_ CD4/CD8 ratio for patients on dual therapy was −0.03 (95% confidence interval (CI) –0.05, –0.0002), and the mean change in CD8 count was +99 (95% CI +12.1, +186.3), taking those on triple therapy as reference. In contrast, there was no evidence for a difference in CD4 count change. When using all counts, there was evidence for a significant difference in the slope of the ratio and CD8 count between people who were switched to triple (points/year change ratio = +0.056, CD8 = −25.7) and those to dual regimen (ratio = −0.029, CD8 = +110.4).

**Conclusions:**

We found an increase in CD8 lymphocytes in people who were switched to dual regimens compared to those who were switched to triple. Patients on monotherapy did not show significant differences. The long-term implications of this difference should be ascertained.

**Electronic supplementary material:**

The online version of this article (10.1186/s12916-018-1046-2) contains supplementary material, which is available to authorized users.

## Background

Long-term side effects of antiretroviral therapy (ART) drugs have led to the introduction in clinical practice of nucleoside reverse transcriptase inhibitor (NRTI)-sparing regimens as double or monotherapy, and their use is now recommended in specific populations according to international guidelines [1, 2]. Indeed, based on the monitoring of surrogate markers of ART efficacy, most of these unconventional regimens, when used in switch studies, have been shown to have a non-inferior virological efficacy and a good CD4 recovery compared to standard triple drug-based therapy [3–8]. The results of these studies were so encouraging that dual combinations are currently being tested in randomized clinical trials of ART-naïve patients [9, 10]. The results of the GEMINI 1-2 international trials on ART-naïve patients with a baseline plasma viremia < 500,000 copies/mL randomized to receive either tenofovir/emtricitabine or lamivudine both combined with dolutegravir are also ongoing [11]. If dual regimes are going to be proved non-inferior to triple combination therapy in the ART-naïve population, a single tablet of this dual regimen will likely be developed. Thus, as a consequence of the publication of the results of these studies, in order to save toxicities and resources, we could see a shift in drug production and clinical use of dual therapies [12, 13]. However, it has been recently shown that virological suppression and CD4 cell count fail to protect from the major causes of death of persons living with HIV (PLWHIV), which are mainly non-AIDS-related events, also known as serious non-AIDS events (SNAEs) [14]. At present, the best marker to evaluate the risk of developing SNAEs has not been determined. Interestingly, the analysis of the data of our and other cohorts have shown that, in contrast with recent data from the Antiretroviral Therapy Cohort Collaboration (ART-CC) [15], a low CD4/CD8 ratio is a predictor of non-AIDS-related events independently from CD4 cell count [16, 17], while other studies have shown an association of this marker with non-AIDS-defining cancers [18] or, more recently, with pulmonary emphysema [19].

Therefore, we hereby aimed to compare CD4/CD8 ratio changes in a group of patients who, in the presence of undetectable plasma HIV viremia, were switched to a protease inhibitor monotherapy (mono) or a dual ART, to a control group who was switched to a standard triple drug-based treatment.

## Methods

### Study design and participants

The Icona Foundation Study is a cohort of HIV-infected patients; this study superseded the original I.Co.N.A. (Italian Cohort of Antiretroviral-Naïve Patients) study (a detailed description of this cohort is given elsewhere [20]), recruiting HIV-positive patients when they are still ART-naïve regardless of the reason. CD4 and CD8 cell counts and viral load are measured every 4–6 months together with other laboratory parameters (e.g., liver and kidney function, lipids, etc.) as well as clinical and therapy data. All patients signed consent forms to participate in the Icona Foundation Study, locally in each of the participating clinical sites, and the research study protocol has been approved by local institutional review boards. The accuracy of non-AIDS events diagnoses is checked by both central and in situ monitoring, by HIV Cohorts Data Exchange Protocol (HICDEP) coding. Central monitoring is done every 6 months to check the accuracy of the data entered. A data monitor goes to each center annually and controls 10–25% of the clinical charts. In case of discordances, the percentage of checked clinical charts increases to 50%, according to the procedure of the D:A:D study, in which Icona participates.

All analyses were restricted to patients in the cohort who did the following: started a combination ART (cART) regimen including three antiretrovirals from being ART-naïve; had reached a confirmed HIV RNA ≤ 50 copies/mL; had switched to either another triple regimen or to double or monotherapy for any reason and at any time after achieving suppression, had maintained virological suppression after 12 months on this same regimen which had been switched to and had in the time window [−3; +3] around 12 months from the switch at least one CD4 and CD8 measurement available (only the first switch episode after achieving suppression was included). Those who switched to triple therapies were considered as the control group in order to have a more uniform population and to avoid, if possible, biases.

If more measurements were available in the defined time window, the nearest to 12 months was selected. We included only patients with a date of switch that occurred after January 2006, because this was the first year in which the switch to mono/dual therapy in people with suppressed HIV RNA was introduced in clinical practice in Italy. We also selected a subgroup of patients for whom at least a CD4 and a CD8 count were available in the window [−3; +3] around 24 months from the switch and the same regimen and the virological suppression were still maintained. Four patients who switched to a dual therapy including maraviroc were excluded from the analyses, as exposure to this drug is believed to increase the CD8 count [21].

The follow-up was interrupted at the date of discontinuation of at least one drug in the regimen started after the switch or at the date of the first of two consecutive HIV RNA levels > 50 or at the date of the CD4/CD8 ratio at 24 months, whichever occurred first.

During the follow-up, the total number of single viral blips, where a blip is defined as (1) a single value of HIV RNA ≥ 50 copies/mL followed by a value < 50 with no treatment switch and (2) a single value of HIV RNA ≥ 200 copies/mL followed by a value < 200, was evaluated and described.

### Statistical analyses

The characteristics of the study population at the time of the switch, stratified by treatment strategy, were compared using the non-parametric Kruskal-Wallis test for continuous parameters and the chi-square test for categorical variables.

Endpoints of the analysis were the change in CD4/CD8 ratio, in CD8, and in CD4 from the switch to 12 and 24 months. Multivariable linear regressions were used to evaluate the association between endpoints and type of regimen, adjusting for main confounders. A linear mixed model with random intercepts and random slopes for repeated measurements of ratio, CD8, and CD4 change was used to compare the course over time of these markers according to regimen strategies. The follow-up started at the date of ART switch and ended at first virological rebound defined by the first of two consecutive HIV RNA measures > 50 copies/mL or a stop/change of at least one drug of the regimen or the date of the last CD4/CD8 count at 24 months from the switch, whichever came first. The sample size of the mono/dual therapy group became too small after 24 months to yield accurate estimates.

The comparison between mono/dual and triple switch was controlled for a number of potential time-fixed confounders measured at the time of the switch or at previous times: age, gender, nation of birth, mode of HIV transmission, hepatitis C virus (HCV) co-infection, AIDS diagnosis, CD4 counts and HIV RNA at ART initiation, years of HIV infection, duration of viral suppression before switch, CD4 and CD8 counts at switch, and reasons for switch. The analysis was not controlled for time-dependent confounders.

## Results

### Study population

A total of 1241 patients were included in the analysis; 1073 switched to triple regimens, 104 to dual regimens, and 64 to monotherapy. Concerning the baseline regimens, almost all patients were receiving tenofovir + emtricitabine, 935 (75.3%), while the third agents are described in Additional file 1: Table S1.

**Fig. 1:**
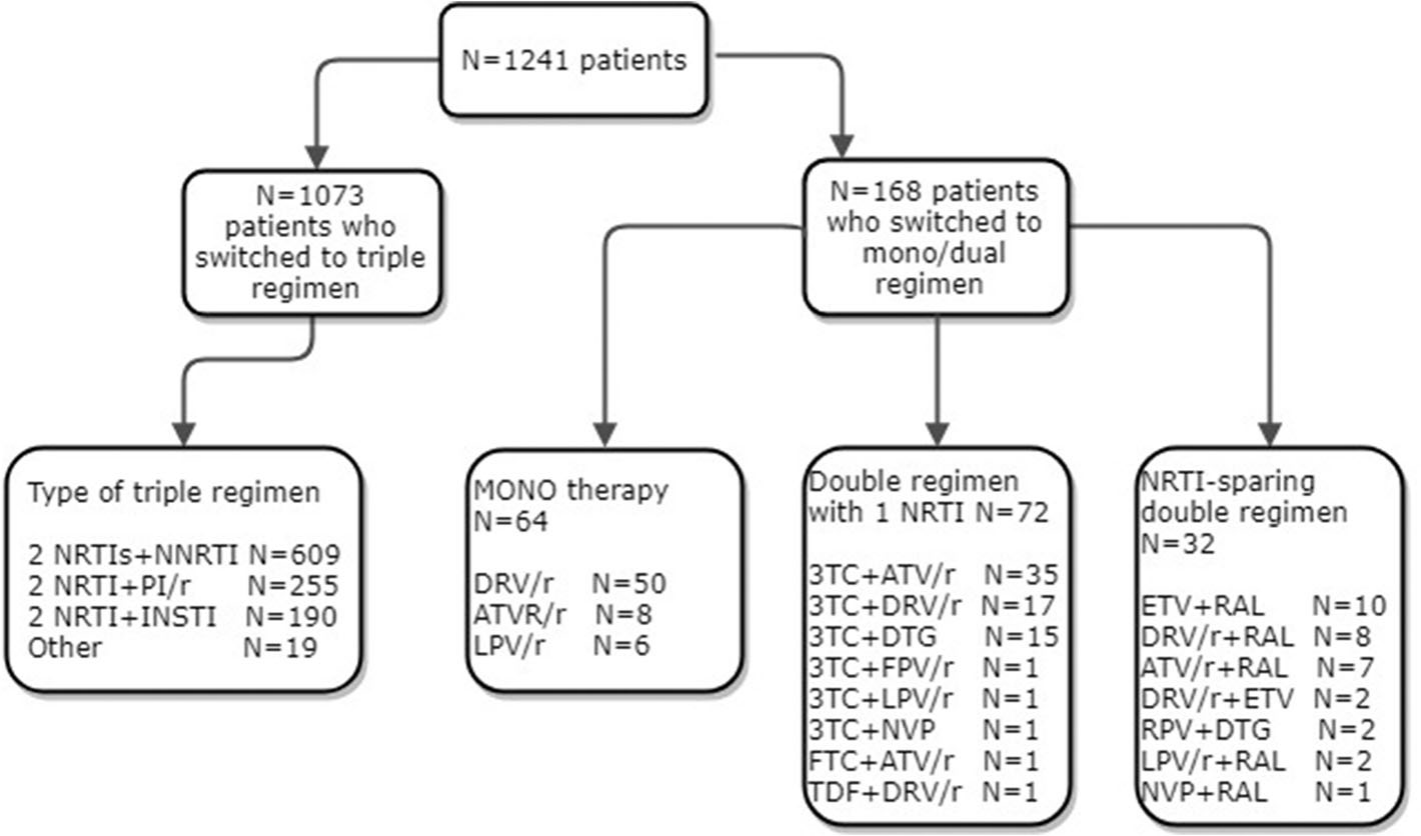
Flow-chart of distribution of the various regimens to which participants were switched

Figure 1 presents a flowchart with the distribution of the various regimens to which participants were switched.

No differences in age, gender, nationality, duration of HIV infection, HCV co-infection, or virological condition at the time of first starting ART were observed among the three groups who switched to triple, mono, or dual therapy (Table 1). The CD4 count before cART initiation was more frequently lower than 350 and less frequently more than 500 cells/uL in patients who switched to a triple regimen compared to those on mono or double therapy. The median duration of the first line regimen and of viral suppression before the switch was similar for the three groups. The CD8 cell counts at the switch were similar, while the median of the CD4/CD8 ratio and the CD4 count at the switch were slightly higher in patients who switched to monotherapy. Patients who were switched to a triple regimen were more likely to have acquired HIV via heterosexual contacts and less frequently through homosexual contacts and were more frequently in Centers for Disease Control and Prevention (CDC) stage C compared to patients with lower drug regimens.

**Table 1:** Main characteristics of study population according to regimen started after switch

	All study population	Triple	Dual	Mono	*p* value
Patients’ characteristics	(*n* = 1241)	(*n* = 1073)	(*n* = 104)	(*n* = 64)	–
Male gender, *n* (%)	988 (79.6%)	853 (79.5%)	86 (82.7%)	49 (76.6%)	0.612
Age, median (IQR)	43 (36–50)	42 (35–50)	45 (38–51)	44 (38–49)	0.065
Mode of HIV transmission, *n* (%)					
Heterosexual	534 (43.0%)	474 (44.2%)	40 (38.5%)	20 (31.3%)	0.025
Injection drug use	100 (8.1%)	92 (8.6%)	5 (4.8%)	3 (4.7%)	
Men who have sex with men	533 (42.9%)	440 (41.0%)	54 (51.9%)	39 (60.9%)	
Other/unknown	74 (6.0%)	67 (6.2%)	5 (4.8%)	2 (3.1%)	
Migrants, *n* (%)	151 (12.2%)	136 (12.7%)	8 (7.7%)	7 (10.9%)	0.317
Previous AIDS event, n (%)	161 (130%)	152 (14.2%)	8 (7.7%)	1 (1.6%)	0.004
Years of HIV infection, median (IQR)	3.4 (1.6–6.3)	3.3 (1.6–6.3)	3.3 (1.6–6.5)	4.2 (2.1–6.1)	0.472
HCV Ab, *n* (%)					
Negative	1057 (85.2%)	908 (84.6%)	88 (84.6%)	61 (95.3%)	0.197
Positive	120 (9.7%)	109 (10.2%)	10 (9.6%)	1 (1.6%)	
Unknown	64 (5.1%)	56 (5.2%)	6 (5.8%)	2 (3.1%)	
CD4 before cART start, *n* (%)					
< 200	368 (29.7%)	334 (31.1%)	24 (23.1%)	10 (15.6%)	0.047
201–350	399 (32.2%)	344 (32.1%)	29 (27.9%)	26 (40.6%)	
351–500	307 (24.7%)	258 (24.0%)	30 (28.9%)	19 (29.7%)	
500+	143 (11.5%)	116 (10.8%)	18 (17.3%)	9 (14.1%)	
Missing	24 (1.9%)	21 (2.0%)	3 (2.8%)	–	
HIV RNA before cART start, *n* (%)					
< 20,000	337 (27.2%)	279 (26.0%)	37 (35.5%)	21 (32.8%)	0.385
20,000–100,000	410 (33.0%)	358 (33.3%)	30 (28.9%)	22 (34.4%)	
100,000–250,000	235 (18.9%)	207 (19.3%)	18 (17.3%)	10 (15.6%)	
250,000+	233 (18.8%)	208 (19.4%)	15 (14.4%)	10 (15.6%)	
Missing	26 (2.1%)	21 (2.0%)	4 (3.9%)	1 (1.6%)	
CD4 at switch, median (IQR)					
< 350	219 (17.7%)	199 (18.6%)	14 (13.5%)	6 (9.4%)	0.008
350–500	285 (23.0%)	255 (23.8%)	23 (22.1%)	7 (10.9%)	
500+	737 (59.3)	619 (57.6%)	67 (64.4%)	51 (79.7%)	
CD4 at switch, median (IQR)	554 (402–740)	547 (394–729)	600 (426–819)	614 (506–807)	0.012
CD8 at switch, median (IQR)	824 (600–1118)	832 (601–1121)	797 (587–1124)	768 (597–1009)	0.467
CD4/CD8 ratio at switch, median (IQR)	0.69 (0.45–0.98)	0.67 (0.44–0.97)	0.78 (0.51–1.10)	0.83 (0.58–1.09)	0.008
CD4/CD8 ratio at switch > = 1, *n* (%)	303 (24.4%)	256 (23.9%)	29 (27.9%)	18 (28.1%)	0.513
Months of antiretroviral exposure, median (IQR)	18 (9–34)	17 (8–35)	18 (12–36)	20 (12–31)	0.184
Months of viral suppression, median (IQR)	21 (11–39)	21 (10–39)	20 (12–37)	20 (13–34)	0.824
Reason for switch, *n* (%)					
Toxicity	330 (26.6%)	294 (27.4%)	27 (26.0%)	9 (14.1%)	< 0.001
Simplification	493 (39.6%)	407 (38.0%)	42 (40.4%)	44 (68.7%)	
Patient’s decision	18 (1.5%)	17 (1.6%)	0 (0.0%)	1 (1.6%)	
Other	387 (31.2%)	346 (32.2%)	31 (29.8%)	10 (15.6%)	
Missing	13 (1.1%)	9 (0.8%)	4 (3.8%)	0 (0.0%)	

The switch to monotherapy compared to those who switched to triple or dual therapy less frequently was made for simplification and more frequently for toxicity, in particular, kidney toxicity (3.8% vs. 14.4%, *p* < 0.001).

The three groups of patients showed a similar length of follow-up after the date of switch (22.1, 21.9, and 22.7 months respectively for the triple, dual, and mono regimens, *p* = 0.084). Over a median follow-up of 22 months (interquartile range (IQR) 21–24) the total numbers of single blips of HIV RNA > 200 and > 50 copies/mL during the follow-up period were similar for the three groups: 7.0% for the triple, 6.7% for the dual, and 5.9% for the mono regimen (*p* = 0.837) and 1.9%, 0%, and 0% (*p* = 0.120) respectively.

#### Univariable analysis: change from baseline to 12 and 24 months

At 12 months from the date of switching, patients on the triple regimen showed a higher mean CD4/CD8 ratio (log_10_) increase +0.12 vs. +0.04 (*p* = 0.017) compared to patients on the dual therapy and +0.07 (*p* = 0.079) compared to patients on the monotherapy (Table 2). We found that 30.6% of patients on the triple regimen had a CD4/CD8 ratio ≥ 1 vs. 36.5% of patients on the dual therapy and 35.9% of patients on the monotherapy (*p* = 0.328).

**Table 2:** Comparison of mean value and standard deviation (SD) of CD4/CD8 ratio, CD8, and CD4 at switch and 12 months after switch between triple and dual and between triple and monotherapy

	Triple (*N* = 1073)	Dual (*N* = 104)	*p* value	Mono (*N* = 64)	*p* value
CD4/CD8 ratio					
At switch, mean (SD)	0.76 (0.46)	0.85 (0.51)	0.054	0.89 (0.50)	0.009
12 months after switch, mean (SD)	0.88 (0.63)	0.89 (0.52)	0.666	0.97 (0.55)	0.091
Change, mean (SD)	+0.12 (0.48)	+0.04 (0.15)	0.004	+0.07 (0.21)	0.079
CD8 cell count					
At switch, mean (SD)	912 (454)	924 (504)	0.778	825 (345)	0.220
12 months after switch, mean (SD)	884 (506)	986 (558)	0.197	860 (410)	0.576
Change, mean (SD)	−28 (471)	+62 (321)	0.010	+34 (222)	0.096
CD4 cell count					
At switch, mean (SD)	588 (289)	652 (320)	0.039	646 (260)	0.022
12 months after switch, mean (SD)	654 (294)	738 (338)	0.012	705 (223)	0.017
Change, mean (SD)	+66 (184)	+86 (180)	0.251	+59 (197)	0.757

The same comparison was performed after restricting to 724 patients who had data at 24 months available (622 triple, 57 dual, and 45 mono), and again in this analysis the triple regimens showed higher mean ratio increase compared to the dual therapy patients (+0.17 vs. +0.08, *p* = 0.024), but not compared to the monotherapy patients (+0.12, *p* = 0.786) (Table 3). However, the percentage of patients with ratio ≥ 1 at 24 months was still not different among patients who switched to triple, dual, and mono regimens (34.4%, 34.3%, 40.0% respectively, *p* = 0.696).

**Table 3:** Comparison of mean value and standard deviation (SD) of CD4/CD8 ratio, CD8, and CD4 at switch and 24 months after switch between triple and dual and between triple and monotherapy in two different regimens in 724 patients who had available data at 24 months

	Triple (*N* = 622)	Dual (*N* = 57)	*p* value	Mono (*N* = 45)	*p* value
CD4/CD8 ratio					
At switch, mean (SD)	0.76 (0.45)	0.80 (0.37)	0.167	0.90 (0.48)	0.025
24 months after switch, mean (SD)	0.93 (0.71)	0.88 (0.43)	0.824	1.02 (0.50)	0.105
Change, mean (SD)	+0.17 (0.58)	+0.08 (0.19)	0.024	+0.12 (0.22)	0.786
CD8 cell count					
At switch, mean (SD)	912 (458)	882 (471)	0.396	812 (363)	0.173
24 months after switch, mean (SD)	867 (451)	911 (411)	0.337	913 (575)	0.870
Change, mean (SD)	−45 (401)	+28 (256)	0.017	+101 (404)	0.012
CD4 cell count					
At switch, mean (SD)	588 (285)	614 (269)	0.402	654 (274)	0.040
24 months after switch, mean (SD)	683 (294)	703 (272)	0.285	795 (332)	0.006
Change, mean (SD)	+95 (181)	+89 (175)	0.943	+141 (229)	0.205

Interestingly, the mean change of CD4 count from baseline to 12 months was not different between the groups: +66 for the switch to triple, +86 for the switch to dual, and +59 for the switch to mono at 12 months and +95, +89, and +141 at 24 months respectively. Conversely, the mean change of CD8 count was different among the three groups. In patients who were switched to dual therapy, at 12 months the mean change was +62 cells/mm^3^, and it was +34 in those who switched to monotherapy, while in the group who switched to triple therapy the mean change was −28 cells. This difference was observed and again was more pronounced at 24 months: patients on the triple regimen had a mean reduction of CD8 of −45 cells, patients switched to the dual regimen presented a mean increase of +28 cells, and there was an increase of +101 for those who switched to the monotherapy.

#### Factors associated with the change of CD4/CD8 ratio and of CD8: simple linear regression analysis of marker change

In the multivariable analysis, the type of switch strategy was associated with the change in CD4/CD8 ratio from the switch to (1) 12 months and (2) 24 months, when analyzed as continuous outcomes (Table 4). After adjusting for age, gender, mode of HIV transmission, nationality, previous AIDS event, years of HIV infection, HCV co-infection, HIV RNA and CD4 at cART initiation, CD4 and CD8 count at switch, reason for switch, and months of viral suppression before switch, patients on dual therapy showed a gain in log_10_ ratio at 12 months and at 24 months lower than that seen in patients on the triple regimen. Patients switched to monotherapy showed a change not significantly different compared to the reference group on triple therapy.

**Table 4:** Two separate multivariable linear models to test the association of the dependent variable with the type of regimen started after switch. Every model is adjusted for: age, gender, mode of HIV transmission, Italian nationality, previous AIDS event, years of HIV infection, HCV co-infection, HIV RNA and CD4 at cART initiation, CD4 and CD8 count at switch, reason for switch, months of viral suppression

a) Multivariable linear regression with changes at 12 months			
	Coefficient	95% CI	*p* value
Dependent variable: CD4/CD8 ratio (log_10_) change at 12 months			
Regimen after switch			
Triple	Ref.		
Dual	−0.03	−0.05, −0.0002	0.049
Mono	−0.007	−0.04, +0.03	0.685
Dependent variable: CD8 change at 12 months			
Regimen after switch			
Triple	Ref.		
Dual	+99.2	+12.1, +186.3	0.026
Mono	+24.7	−84.1, +133.5	0.656
Dependent variable: CD4 change at 12 months			
Regimen after switch			
Triple	Ref.		
Dual	+28.8	−8.0, +65.6	0.125
Mono	−4.8	−50.8, +41.1	0.837
b) Multivariable linear regression with changes at 24 months			
	Coefficient	95% CI	*p* value
Dependent variable: CD4/CD8 ratio (log_10_) change at 24 months			
Regimen after switch			
Triple	Ref.		
Dual	−0.06	−0.10, −0.02	0.003
Mono	−0.01	−0.06, +0.03	0.549
Dependent variable: CD8 change at 24 months			
Regimen after switch			
Triple	Ref.		
Dual	+192.0	−8.4, +392.3	0.060
Mono	+29.3	−200.7, +259.4	0.802
Dependent variable: CD4 change at 24 months			
Regimen after switch			
Triple	Ref.		
Dual	+2.1	−48.9, +53.1	0.936
Mono	+51.2	−7.3, +109.8	0.086

From multivariable models with the change of CD8 at 12 and 24 months as the outcome, patients on dual therapy showed a higher mean change than that seen in people on triple therapy. Patients on monotherapy showed a mean change of CD8 not different compared to that for subjects on the triple regimen. No difference was found for the CD4 change between the two types of regimen.

#### Mixed models

Our 1241 patients contributed 6528 CD4/CD8 ratio measurements over a period of 24 months. A median number of 5 (IQR 4–7) CD4/CD8 ratio values/patient were recorded. In the univariable analysis, the estimate of the overall increase in the log_10_ ratio was +0.040 points/year (95% confidence interval (CI) +0.036, +0.044; *p* < 0.001). Points/year change in the log_10_ ratio were +0.042 (95% CI +0.037, +0.047) for patients on the triple regimen and –0.013 (95% CI –0.029, −0.003) for subjects who switched to dual therapy (Table 5). After adjustment for baseline values in the intercept, changes for the triple regimen were +0.056 (95% CI +0.047, +0.064), while for the dual regimen they were −0.029 (95% CI –0.056, –0.002). There was evidence for a significant difference in slope between the triple and dual regimens (interaction test, *p* = 0.033).

**Table 5:** Unadjusted and adjusted coefficients from linear mixed model regression analysis to test the association between the dependent variable and type of regimen started after switch. Every model is adjusted for: age, gender, mode of HIV transmission, Italian nationality, previous AIDS event, years of HIV infection, HCV co-infection, HIV RNA and CD4 at cART initiation, CD4 and CD8 count at switch, reason for switch, months of viral suppression

	Unadjusted coefficient	95% CI	Adjusted coefficient	95% CI
CD4/CD8 ratio (log_10_)				
Regimen after switch				
Triple	+0.042	(+0.037, +0.047)	+0.056	(+0.047, +0.064)
Dual	−0.013	(−0.029, −0.003)	−0.029	(−0.056, −0.002)
Mono	−0.013	(−0.030, +0.005)	−0.011	(−0.044, +0.021)
CD8, cells/mm^3^				
Regimen after switch				
Triple	−22.2	(−48.0, +3.7)	−25.7	(−51.6, +0.28)
Dual	+114.9	(−32.9, +197.0)	+110.4	(+27.3, +193.6)
Mono	+73.5	(−25.4, +172.5)	+64.7	(−35.6, +165.0)
CD4, cells/mm^3^				
Regimen after switch				
Triple	+46.7	(+40.3, +53.1)	+45.2	(+30.7, +59.6)
Dual	+7.6	(−13.5, +28.8)	+32.1	(−13.1, +77.3)
Mono	+23.6	(−1.40, +48.7)	+18.0	(−36.9, +72.9)

The overall observed trend for CD8 count was −7.2 cells/year (95% CI –31, +17; *p* = 0.559). In the univariable analysis, subjects on the triple regimen showed a change of −22.2 (95% CI –48.0, +3.7) CD8 cells/year, while patients on dual therapy had a mean change of +114.9 (95% CI –32.9, +197.0) CD8 cells/year. In the multivariable analysis the CD8 counts of patients on the triple regimen were reduced by −25.7 (95% CI –51.6, +0.28), while CD8 counts for the dual regimen showed a significant increase of +110.4 cells/year (95% CI +27.3, +193.6). There was evidence for a significant difference in slope between the triple and dual regimens (interaction test *p* = 0.009). Neither the CD4/CD8 ratio nor the CD8 count showed a significant linear trend in the group of patients on monotherapy.

## Discussion

The main result of our study is that when comparing three groups of patients undergoing different switch strategies in the presence of undetectable HIV RNA, those who were switched to dual regimens showed a stabilization of the CD4/CD8 ratio, while the CD4/C8 ratio of those who were switched to a three-drug-based regimen continued to improve after the switch. This result appears not to be due to the CD4 increase, since no significant difference in the CD4 count trajectory between the two groups could be detected, but to a specific increase in CD8 lymphocyte count in participants switching to dual therapy. More importantly, because CD4 count continued to increase and plasma viral load continued by analysis design to be undetectable in all subjects, this difference in the ratio would possibly go undetected by standard monitoring of HIV RNA and CD4 count alone.

A lower CD4/CD8 ratio can be interpreted as a measure of dysregulation of a patient’s immune system, known as immunological risk phenotype, in the general population and has been clearly associated with a higher risk of AIDS and non-AIDS events in HIV-infected patients [16, 17]. Therefore, more and more frequently, in people with undetectable HIV RNA, chronic inflammation is becoming an important long-term prognosis issue, and measuring the CD4/CD8 ratio could clinically represent a reliable tool to monitor this phenomenon.

Indeed, despite successful cART, there is evidence for continuous quantitative, qualitative, and functional defects in the CD8 compartments, although some of these defects in some cases could be reversed by early treatment [22]. However, during chronic HIV infection, peripheral CD8 T cells persistently maintain several defects which are reflected in continuous maintenance of the immune activation parameters. It has been shown that this activation contributes to immunologic exhaustion, hyporesponsiveness of specific T cells, and perturbations in the T cell receptor repertoire. However, reasons for the persistence of elevated CD8 T cells during treatment have not been fully elucidated. Long-term therapy usually determines a significant CD4 recovery, contrasting with, despite a decrease from baseline, persistently elevated CD8 T cell counts [23]. Previous analyses of cohort data have shown that elevated CD8 T lymphocytes at cART initiation were associated with a poor increase in the CD4 T cell count, even if the studies showed no data on CD8 activation [22, 23].

Indeed, in Caby’s study, 50% of individuals with a ratio < 1 despite a normalized CD4 count (> 500 cells/uL) still displayed a CD8 count that remained abnormally high (> 1000 cells/uL) [24]. Moreover, only individuals with a ratio ≥ 1.5 achieved an apparently normal median CD8 count when compared to healthy HIV-seronegative individuals [24]. Furthermore, after 8 years of suppressive cART, only one-third of patients of the French Hospital Database on HIV cohort achieved CD4/CD8 restoration [23]. Encouragingly, Saracino et al. have shown that patients with more than 15 years of cART had a progressive increase in the CD4/CD8 ratio which never reached a plateau, but patients included in that analysis were receiving triple therapy [25]. Our data suggest that treating patients with less than three drugs might lead to a stop of this virtuous trend.

What could explain the CD8 increase observed in our patients who were switched to dual therapies? One possible explanation is that two drugs could not suppress HIV as well as three drugs can. A small residual viremia, probably in lymphoid tissues, could thus trigger the production of proinflammatory and/or homeostatic cytokines that, in turn, would stimulate and/or maintain CD8 T cell proliferation/activation. In our analysis, everybody had an HIV RNA ≤ 50 copies, but we could not create a finer classification below this threshold with the available information on the assays used. In addition, we have shown that viral blips > 50 copies/mL were rare, and their rate of occurrence was similar between the mono, dual, and triple regimen groups, while those > 200 copies/mL were actually more frequent among those who were in the triple regimen. Moreover, it has been previously described that even more sophisticated markers of residual HIV replication, such as HIV -DNA, did not differ between patients switched to dual or monotherapy compared to those continuing triple therapy [26, 27]. However, it is possible that viral replication restarts in lymphoid tissues, and it could be detected by new sophisticated techniques like digital droplet polymerase chain reaction (PCR) or functional virological assays [28] or by analyzing cells resident in those anatomic districts—assays that typically are not available in routine clinical studies. Indeed, recent data from the Collaboration of Observational HIV Epidemiological Research in Europe (COHERE) have shown that, in HIV controllers, the decrease of the ratio and the increase in CD8 lymphocyte counts preceded by 5 years the end of virological control. Moreover, CD8 lymphocyte counts increased significantly in those who experienced loss of virological control, whereas they remained stable in the other groups [29]. Currently it is very unlikely to see randomized clinical trials which last longer than 144 weeks, which is too short a time to verify the COHERE observation in the context of randomized data. Moreover, very rarely are CD8 lymphocyte count data reported in clinical trials. Helleberg et al. have shown that in patients receiving cART for 10 years a value of CD8 lymphocyte count which stays above 1500 cells/uL is associated with increased non-AIDS-related mortality (mortality rate ratio 1.82; CI 1.09–3.22) [22].

The changes in ratio and in CD8 were not detected in subjects switched to monotherapy. Indeed, although there was a trend in the univariable analysis, it was not confirmed with the multivariable linear regression and mixed models analysis. One possible explanation could be the lower number of subjects who were switched to monotherapy, but another could be that their better immunological profile was definitively better at baseline.

We are well aware of the limitations of our study. First, among dual therapies we could not examine protease inhibitor (PI) and integrase strand transfer inhibitor (INSTI)-based combinations separately (not many people received dolutegravir either); second, a median follow-up of 2 years could not allow us to evaluate either the persistence of this phenomenon or the impact of the strategies on the onset of non-AIDS events; third, we did not have more detailed immunological data than the counts themselves, and it would be extremely relevant to stratify the analysis according to specific CD8 subpopulations and CD38/HLDR+ expression. Finally, since our analysis is retrospective and non-randomized, it only accounts for measured fixed confounders at the time of the therapy switch. Thus, we cannot rule out bias being introduced by unmeasured confounders or time-dependent confounders that have not been appropriately controlled for. In particular, even in the controlled setting of undetectable HIV RNA, patients switching to a reduced drug regimen (less than three) instead of another three-drug regimen might be determined by the factors that we did not measure.

After more than 20 years since the introduction of cART because of the use in clinic of new potent and better tolerated drugs, there is now interest in reassessing the ideal number of antiretroviral drugs which need to be prescribed. In the absence of data from randomized studies, our results appear to be relevant to this debate. Current research on the clinical effectiveness of dual cART regimens is focused on non-inferiority of the virological outcome and on saving toxicity due to reducing drugs in the regimens. Nevertheless, it is possible that dual regimens have an unfavorable impact on the CD4/CD8 ratio, and this possibility must be thoroughly investigated before implementing novel drug treatment strategies including less than three drugs.

At present, there is evidence supporting CD8 count monitoring as optional in patients with satisfactory virological and immunological control during ART [30], and this is currently taken into account by international guidelines [2]. However, immunological monitoring based only on the predictive role of a low CD4 count on the risk of developing clinical events may underestimate the role of the CD8 count as a surrogate of a proinflammatory state.

## Conclusions

In this cohort of treated and virologically suppressed HIV-positive patients, the CD4/CD8 ratio continued to increase in those who switched to triple regimens, whereas it stabilized due to a selective increase in CD8 cells among those who switched to dual therapy. Our results suggest that, before dual therapy may eventually become a standard of care in patients with HIV infection, the impact of this strategy on the immune system should be further assessed and considered.

## Additional file

Additional file 1: **Table S1.** Baseline third drugs in patients who switched to triple, dual, or monotherapy. (DOCX 12 kb)

## Abbreviations

ART: antiviral therapy
cART: combination antiviral therapy
NRTI: nucleoside reverse transcriptase inhibitor
SNAEs: serious non-AIDS events
ART-CC: Antiretroviral Therapy Cohort Collaboration
I.Co.N.A.: Italian Cohort of Antiretroviral-Naïve Patients
HICDEP: HIV Cohorts Data Exchange Protocol
CDC: Centers for Disease Control and Prevention
