# Supplementary material for: Switching to dual/monotherapy determines an increase in CD8+ in HIV-infected individuals: an observational cohort study
Source: BMC Med. 2018 May 29;16:79. doi: 10.1186/s12916-018-1046-2 (PMC5972434; doi:10.1186/s12916-018-1046-2)
Supplement: Supplementary file 1 — Table S1. Baseline third drugs in patients who switched to triple, dual, or monotherapy. (DOCX 12 kb) [file 12916_2018_1046_MOESM1_ESM.docx]

|  | **Triple** | **Dual** | **Mono** |
| --- | --- | --- | --- |
| **Patients’ characteristics** | **(n=1073)** | **(n=104)** | **(n=64)** |
| 2NRTI+NNRTI  EFV  NVP  RPV | 425 (39.6%)  372  29  24 | 16 (15.4%)  7  2  7 | 8 (12.5%)  6  0  2 |
| 2NRTI+PI/r  DRV/r or c  ATV/r  LPV/r | 562 (52.4%)  201  184  177 | 75 (72.1%)  23  43  9 | 54 (84.4%)  37  9  8 |
| 2NRTI+INSTI | 70 (6.5%) | 9 (8.7%) | 1 (1.6%) |
| RGV | 35 | 3 | 0 |
| DGV | 24 | 3 | 0 |
| EVG | 11 | 3 | 1 |
| Only NRTIs | 6 (0.6%) | 0 | 0 |
| Other | 10 (1.0%) | 4 (3.9%) | 1 (1.6%) |
